# Fabrication of Cellulose-Based Hydrogels Through Ionizing Radiation for Environmental and Agricultural Applications

**DOI:** 10.3390/gels11080604

**Published:** 2025-08-02

**Authors:** Muhammad Asim Raza

**Affiliations:** School of Chemical Engineering, Yeungnam University, Gyeongsan 38541, Republic of Korea; muhammadasimraza@yu.ac.kr

**Keywords:** cellulose, ionizing radiation, hydrogels, environmental remediation, agricultural applications

## Abstract

Hydrogels exhibit remarkable physicochemical properties, including high water absorption and retention capacities, as well as controlled release behavior. Their inherent biodegradability, biocompatibility, and non-toxicity make them suitable for a wide range of applications. Cellulose, a biodegradable, renewable, and abundantly available polysaccharide, is a viable source for hydrogel preparation. Ionizing radiation, using electron-beam (EB) or gamma (γ) irradiation, provides a promising approach for synthesizing hydrogels. This study reviews recent advancements in cellulose-based hydrogels, focusing on cellulose and its derivatives, brief information regarding ionizing radiation, comparison between EB and γ-irradiation, synthesis and modification through ionizing radiation technology, and their environmental and agricultural applications. For environmental remediation, these hydrogels have demonstrated significant potential in water purification, particularly in the removal of heavy metals, dyes, and organic contaminants. In agricultural applications, cellulose-based hydrogels function as soil conditioners by enhancing water retention and serving as carriers for agrochemicals.

## 1. Introduction

A hydrogel is a cross-linked network of hydrophilic polymers that are capable of absorption and retention of large amounts of water or aqueous solutions without compromising structural integrity [1,2,3]. Owing to their unique characteristics, such as high water content, softness, biocompatibility, and flexibility, hydrogels are widely applied in diverse fields, including tissue engineering, drug delivery, biosensors, metal ion recovery, environmental remediation, and agriculture [4,5,6,7]. Although synthetic polymers are commonly used in hydrogel fabrication, growing research interest has shifted toward renewable alternatives, such as peptides, amino acids, and polysaccharides, which are environmentally sustainable and cost-effective [8,9,10]. Among these, cellulose and its derivatives have been increasingly utilized in various applications due to their biodegradability and physicochemical properties [11,12].

The use of ionizing radiation technology for hydrogel fabrication is considered environmentally friendly, as it minimizes byproduct formation and reduces the risk of contamination [13]. When polymeric materials are exposed to ionizing radiation, they generate ions, excited states, free radicals, and highly reactive intermediates. These intermediates can undergo various reaction pathways, leading to imbalances, reconfigurations, or the formation of new bonds [14,15,16,17].

The increase in wastewater generation is primarily attributed to unplanned urbanization, rapid industrialization, various anthropogenic activities, and inadequate waste management practices. Industrial effluents typically contain heavy metals, dyes, and organic contaminants, all of which pose serious threats to sustainable ecosystems [18,19,20,21]. In living organisms, heavy metal ions such as copper (Cu(II)), lead (Pb(II)), nickel (Ni(II)), cadmium, and arsenic tend to accumulate. The absence of degradability, high permeability, and toxicity causes several physiological disorders that lead to altered development and pose a serious risk to human health [22,23]. Even at low concentrations, dyes can impede light penetration in water bodies, significantly reducing dissolved oxygen levels and endangering aquatic life. In many cases, the presence of dyes leads to anaerobic digestion that generates carcinogenic compounds, which can bioaccumulate and enter the food chain through aquatic organisms [24]. Organic compounds, such as personal care items, pharmaceuticals, endocrine-disrupting substances, and pesticides, are a significant category of wastewater pollutants [25]. These are particularly risky because traditional water treatment methods often fail to break down or effectively remove some of these organic compounds [26]. Consequently, trace amounts of these organic contaminants are frequently found in natural water bodies, treated wastewater, and processed drinking water [27].

Among various wastewater treatment methods, adsorption is considered the most effective and widely applied due to its operational simplicity, cost-effectiveness, and lack of harmful by-products [28,29]. Hydrogels, which are three-dimensional polymer networks, have garnered significant attention for wastewater treatment owing to their high contaminant removal efficiency [30,31]. Specifically, cellulose-based hydrogels demonstrate strong potential for environmental remediation due to their affordability, hydrophilicity, biocompatibility, biodegradability, and non-toxicity [32].

The demand for eco-friendly and sustainable solutions that can enhance crop yields while mitigating the environmental impact of current agricultural practices is steadily increasing. Conventional soil conditioners and fertilizers often contribute to soil degradation, nutrient leaching, and environmental pollution. Therefore, developing new strategies that promote sustainable agriculture and effectively address these challenges is essential. Climate change—characterized by increased drought, reduced rainfall, and temperature fluctuations—has exacerbated these issues, leading to decreased crop yields due to water loss from runoff and evaporation. Consequently, advanced and immediate interventions are required to mitigate these adverse effects and support the development of resilient farming practices [33,34,35]. Hydrogels offer a promising alternative in this regard. Their porous structure and hydrophilic nature enable them to retain substantial amounts of water. In recent decades, natural polymers have been extensively employed in the synthesis of agricultural hydrogels. These hydrogels are biodegradable, non-toxic, and biocompatible, making them suitable for use as soil conditioners. Cellulose-based hydrogels improve soil water retention, alleviating water stress in crops grown in arid or drought-prone regions. Additionally, their superior transport capabilities facilitate the efficient and controlled release of agrochemicals while also contributing to soil pH regulation [36,37].

This review systematically examines the current state of research on the fabrication of cellulose-based hydrogels using ionizing radiation. It explores the structural features, synthesis methodologies, and applications of these hydrogels, as well as the potential benefits and challenges associated with this fabrication technique. Furthermore, the review aims to provide insights into the versatility and efficacy of cellulose hydrogels in both environmental (i.e., the removal of heavy metals, dyes, and organic contaminants) and agricultural (i.e., soil conditioning and controlled release of agrochemicals) applications.

## 2. Cellulose and Its Derivatives

Cellulose is primarily derived from plants, while other sources, such as microorganisms and tunicates (marine organisms), are less commonly used. It is composed of glucose molecules connected by β-glycosidic bonds to create the cellulose polymer [38]. As a natural, abundant, and renewable material, cellulose offers numerous advantages, including low toxicity, biodegradability, and a large surface area. Additionally, it contains a substantial number of surface functional groups, such as carboxylic and hydroxyl groups, that can be modified, combined with organic compounds, or enhanced with additional functional groups for specific purposes [14].

Bacterial cellulose (BC) is a form of cellulose, synthesized by *Acetobacter xylinum*, is a natural polymer of significant interest due to its unique structural and biochemical properties, including bioadaptability, an ultrafine nanofibrous network, non-toxicity, and chemical stability [39]. The uniform and ultrafine fibrous structure of BC imparts excellent mechanical properties and water absorption capacity, including a high elastic modulus and tensile strength [40]. These beneficial characteristics make BC a promising candidate for the development of advanced adsorptive materials, such as hydrogels, for drug delivery applications.

**Figure 1:**
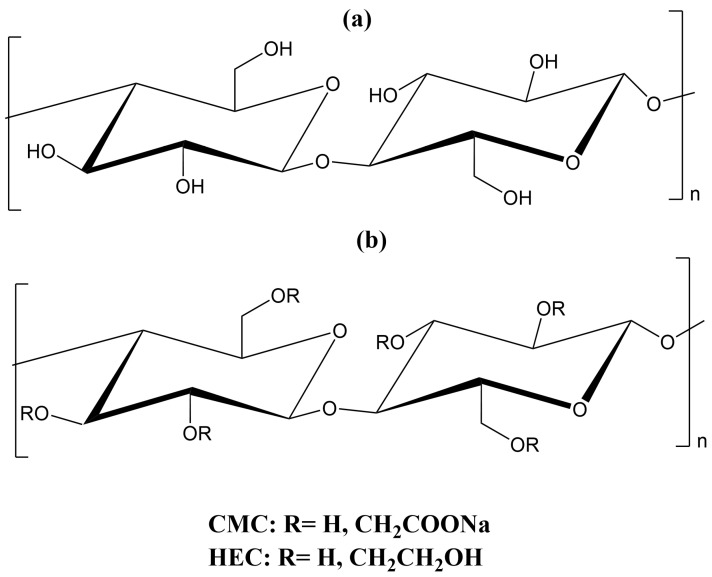
Chemical structure of (**a**) BC and (**b**) cellulose derivatives.

Several derivatives of cellulose, such as carboxymethyl cellulose (CMC) and hydroxyethyl cellulose (HEC), have been widely employed in environmental and agricultural applications. CMC, a water-soluble anionic cellulose derivative, has attracted significant attention in the development of superabsorbent hydrogels due to its negatively charged carboxylate groups distributed along the cellulose backbone. These functional groups enhance the material’s capacity to absorb water and facilitate the formation of hydrophilic hydrogels [15]. HEC, a derivative of cellulose ether, is extensively utilized in both industrial and biomedical fields owing to its availability, affordability, water solubility, and biocompatibility [41]. The chemical structure of BC and cellulose derivatives is shown in Figure 1.

Cellulose, BC, and CMC are compared in various aspects such as source, synthesis route, solubility in water, availability, solubility in water, mechanical properties, toxicity, and applications [42,43]. While Table 1. shows the comparison of characteristics for cellulose/BC/CMC.

## 3. Ionizing Radiation

Ionizing radiation is a highly efficient, versatile, and environmentally friendly technique for synthesizing innovative materials and modifying polymers, especially polysaccharides, which are non-toxic, renewable, and biodegradable natural polymers. Unlike conventional synthesis methods, this approach enables material processing in various physical forms at suitable temperatures, often at room temperature, without the need for initiators or additives. Predominantly, this technology utilizes the high ionization energy radiation in the from gamma (γ) rays or beams of electrons to create cross-link networks from monomers or polymers in aqueous conditions. Moreover, the process is uniform, highly reliable, and waste-free, offering high productivity with minimal processing time [44,45]. Additionally, this technique allows precise control over the degree of cross-linking by adjusting the radiation dose or polymer concentration. It also produces high-purity hydrogels with a higher swelling capacity compared to those synthesized by conventional methods [46].

There are two types of ionizing radiation used for the preparation of hydrogels. γ-irradiation causes ionization in polymer chains, leading to chain breaking and cross-linking through a process involving free radicals. The degree of cross-linking depends on factors such as its phase structure, the polymer’s composition, the duration of radiation exposure, the dosage, and the properties of the radiation source [47]. Electron-beam (EB) irradiation is another effective method for synthesizing hydrogels, as it employs direct electrons to initiate radical reactions without the need for initiators, enabling the cross-linking process to occur more rapidly [48]. Both methods have been compared in terms of various parameters to fully understand them [49]. Table 2 shows a comparison between γ and EB irradiation.

## 4. Fabrication of Cellulose-Based Hydrogels Using Ionizing Radiation

There are three synthesis routes for cellulose-based hydrogels, including physical, chemical cross-linking, and ionizing radiation-induced copolymerization and grafting [50]. In chemical cross-linking, the cellulose chains are covalently linked through the utilization of chemical reagents [51]. In physical cross-linking, cellulose chains are crosslinked using physical forces like pressure or temperature [52].

**Figure 2:**
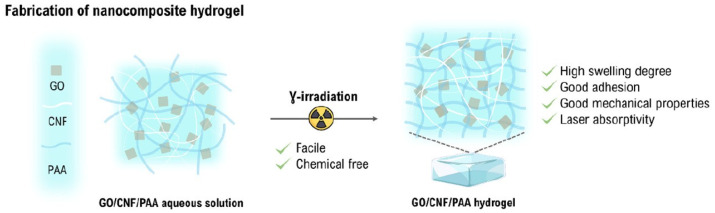
γ-irradiation induced cross-linking of GO/CNF/PAAC aqueous solution to develop a hydrogel. Reproduced from [54] with permission from the Elsevier publishers.

Ionizing radiation has emerged as a favorable technology for the preparation of cellulose-based hydrogels [46]. Cellulose has been formulated in the form of blends with other polymers, monomers are grafted on the cellulose backbone, act as reinforcement in the nanostructural shape, and cellulose derivatives with other nano-reinforcements can develop nanocomposites through ionizing radiation. In this regard, Wach et al. first started radiation-induced cross-linking of polysaccharides to cross-link CMC with different degrees of substitution. Afterwards, this method became widely accepted for the fabrication of hydrogels from other types of polysaccharides as well. This technique has certain advantages, like the sterilization of products, and the polysaccharide-based hydrogels are preferred over synthetic hydrogels due to the biodegradability and origin of the substrate [53]. Whereas, Passornraprasit et al. prepared γ-irradiation cross-linked graphene oxide (GO) and cellulose nanofiber (CNF) embedded poly(acrylic acid) (PAAC) hydrogels under a nitrogen atmosphere [54]. Reactive species are generated on the polymeric chain upon exposure to γ-irradiation. Hydrogen abstraction and radical combination reactions assist in the formation of a cross-linked polymer network structure. The developed hydrogels showed high swelling, adhesion, laser absorptivity, and mechanical properties, as shown in Figure 2.

**Figure 3:**
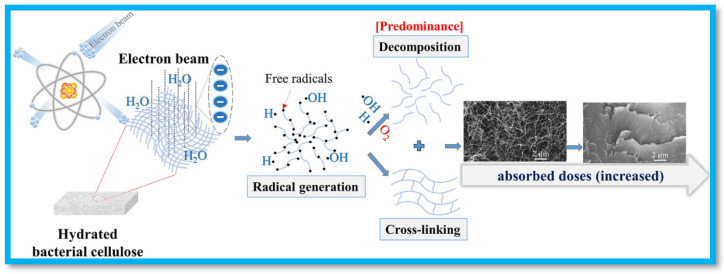
EB-assisted cross-linking/decomposition of hydrated BC sheets to form aerogel-like morphology. Reproduced from [45] with permission from Springer Nature Link publisher.

In the case of EB-irradiation, Sung et al. developed CMC and carbon materials-based nanocomposite superabsorbent (SAB) hydrogels using EB-induced polymerization and solution polymerization [55]. Carbon materials such as activated carbon, GO, and reduced graphene oxide (rGO) were used as fillers. Direct electrons generated free radicals on the polymer and additives, which made linkages between them to develop a cross-linked structure. The EB-irradiation induced hydrogels showed superior physicochemical properties than solution polymerization-generated hydrogels. Similarly, Saiki et al. synthesize CMC hydrogel using the EB-irradiation technique. The results revealed that ·OH radicals resulting from water radiolysis are responsible for the cross-linking of CMC. Furthermore, they reported that the reaction of the ·OH radical created CMC radicals in aqueous solution [44]. In another work, Phan et al. tailored characteristics of BC into aerogel-like morphology using EB-irradiation-assisted cross-linking/decomposition [45]. The mechanism of modification is shown in Figure 3, where water molecules undergo free radical generation through exposure to EB-irradiation. The stable macroradicals undertake a cross-linking reaction, and a parallel decomposition phenomenon is also reported in the literature.

## 5. Environmental Applications

This chapter highlights the application of ionizing radiation-processed cellulose-based hydrogels in environmental remediation. A comprehensive summary of these environmental applications is presented in Table 3.

### 5.1. Water Purifications

Wastewater purification represents a critical application area for polymer-based hydrogels. The term “wastewater” encompasses effluents from industrial, domestic, and agricultural sources [56,57,58]. These effluents typically contain a wide range of pollutants that pose both direct and indirect threats to human health, particularly when such contaminants enter the food chain. Among the most hazardous pollutants are heavy metals, dyes, and organic compounds. In this regard, Zhang et al. fabricated a phosphorylated cellulose-based hydrogel featuring both physical and chemical cross-linking to remove uranium (U(VI)) from aqueous solution [59]. This hydrogel was synthesized using a combination of γ-irradiation technique and phytic acid modification to enhance the phosphorylated functionalization of cellulose. The dual cross-linking strategy significantly improved the hydrogel’s mechanical integrity, yielding a strain of 49.20% and a compressive strength of approximately 0.13 MPa. TGA results showed that prepared hydrogels had excellent thermal stability. Cross-linking of cellulose hydrogels was confirmed by the spectroscopic analyses. Adsorption studies revealed that the combined effect of carboxy and phosphate functional groups markedly enhanced the hydrogel’s capacity to adsorb U(VI). The maximum adsorption capacity (Q_max_) for U(VI) was 449.5 mg/g, and the hydrogel maintained a desorption rate of 87.1% after five cycles of reuse. The adsorption isotherm data fit well with the Langmuir model. The chemisorption phenomenon dictated the adsorption of adsorbate, suggesting a pseudo-second-order (PSO) model. This work addresses the drawbacks of conventional cellulose-based adsorbents and provides an efficient and cost-effective adsorbent for U(VI) removal.

Excessive fluorine (F) levels in zinc (Zn) hydrometallurgical systems and aquatic environments pose a pressing environmental challenge. In response, Zeng et al. prepared zirconium oxychloride (ZrOCl_2_)-immobilized CMC/multiwalled carbon nanotubes (MW-CCNTs)-based hydrogels using the EB-irradiation technique for efficient F removal [60]. The FTIR analysis confirmed the successful immobilization of ZrOCl_2_ on CMC/MW-CCNTs, and TGA analysis proved thermal stability. SEM analysis confirmed that the incorporation of MW-CCNTs provides a nanotubes/fiber network structure and increases the gel fraction (%) of CMC/ZrOCl_2_ hydrogels. Kinetics studies showed that adsorption occurred through a chemical phenomenon, suggesting that it conformed to the PSO kinetic model. The isotherm studies revealed that adsorption data fit well with both the Freundlich and Langmuir models. The Q_max_ of the hydrogels was 36.657 mg/g. The selectivity coefficients of the hydrogel for fluoride (F^−^) over phosphate (PO_4_^3−^), nitrate (NO_3_^−^), and sulfate (SO_4_^2−^) ions were 140.34, 72.572, and 10.953, respectively. This material also demonstrated strong potential as a selective adsorbent for F from high-concentration SO_4_^2−^ solution. Accordingly, this design provides a promising adsorbent for the selective removal of F from both hydrometallurgical systems and water bodies.

The water purification section is further categorized based on the removal of toxic heavy metals, dyes, and organic contaminants.

#### 5.1.1. Removal of Heavy Metals

Among the various pollutants found in wastewater, heavy metals are of particular concern due to their toxicity, persistence, and bioaccumulative nature. Consequently, the removal of heavy metals has become a critical focus in wastewater treatment research [61]. Numerous studies have demonstrated that ionizing radiation-processed cellulose-based hydrogels exhibit strong potential for the adsorption of heavy metal ions. In this context, Badawy et al. developed acrylic acid (AAC)- and HEC-based hydrogels using γ-irradiation and subsequently modified them with cationic and amphoteric surfactants to enhance their efficacy for Pb(II) removal [62]. Spectroscopic studies showed the interaction of constituents. SEM analysis confirmed the even distribution of the surfactants and the porous structure of the PAAC/HEC hydrogel. EDX studies proved the incorporation of the surfactant within the polymeric matrix. Batch adsorption tests confirmed that each hydrogel formulation effectively adsorbed Pb(II) ions. Optimal adsorption occurred under conditions of pH 5, contact time of 240 min, and solution volume-to-mass ratio of 0.4 L/g. To evaluate the hydrogels’ selectivity toward Pb(II), competitive adsorption experiments were conducted using solutions containing Zn(II), Pb(II), and Co(II) ions. The adsorption behavior of Pb(II) was analyzed using adsorption isotherms and kinetic modeling. The data were well described by the pseudo-first-order (PFO) kinetic model and the Freundlich isotherm. The Q_max_ for Pb(II) was 26.65 mg/g. Additionally, the hydrogels exhibited temperature responsiveness, with the highest removal efficiency of 97% observed at 65 °C. Thermodynamic parameters showed that the adsorption process was spontaneous and endothermic.

**Figure 4:**
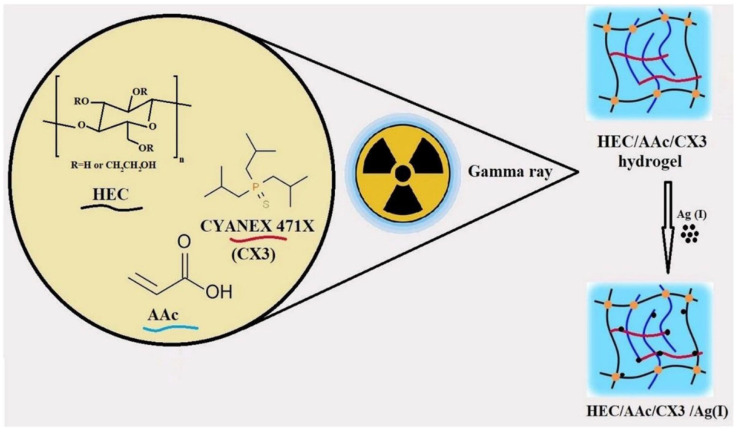
Radiation-induced synthesis of CYANEX 471X/AAC/HEC hydrogels for Ag(I) ions capture. Reproduced from [63] with permission from Springer Nature Link publisher.

In another study, Masry et al. synthesized triisobutylphosphine sulfide (CYANEX 471X)/AAC/HEC-based hydrogels via γ-irradiation induced polymerization for the removal of silver (Ag(I)) ions from an acidic nitrate medium, as shown in Figure 4 [63]. SEM micrographs revealed that the prepared hydrogels before adsorption have a porous structure, while after adsorption pore size decreased owing to the penetration of Ag(I) metal ions. The XRD pattern confirms the sorption of silver ions after adsorption, and the presence of silver content was proved with EDS studies. The results indicated that the developed hydrogels exhibited non-Fickian diffusion, confirming that polymer chain relaxation and solvent diffusion occurred at comparable rates, thus making them suitable for further Ag^+^ ion sorption. A maximum desorption efficiency of 70% was achieved after one desorption cycle with 0.5M ammonium thiocyanate, and the Q_max_ reached 12 mg/g after five sorption cycles. Thermodynamic parameters showed that the adsorption process was non-spontaneous and exothermic. Additionally, the hydrogels demonstrated high selectivity toward Ag(I) ions, with facile desorption steps and reusability cycles.

Furthermore, Wang et al. developed polysaccharide-based adsorbents, including hydroxypropyl methylcellulose phthalate (HPMCP), carboxymethylated chitosan (CM-CS), and CMC, using γ-irradiation for the adsorption and desorption of strontium (Sr(II)) ions [64]. XPS analysis reveals that Sr(II) ions are chemically adsorbed into the polysaccharide-based adsorbents through ion exchange on the carboxyl groups. The adsorption capacity increased with decreasing ionic strength and rising pH. Maximum adsorption was achieved within 2 h for HPMCP, 0.5 h for CM-CS, and 1 h for CMC. The adsorption behavior of Sr(II) ions closely followed the Langmuir isotherm model. The Q_max_ was 83.3 mg/g for HPMCP, 99.0 mg/g for CM-CS, and 108.7 mg/g for CMC. The desorption of Sr(II) ions was achieved by heating the adsorbents in a moist hot environment or by immersing them in solutions with high ionic strength or low pH.

Similarly, Gad et al. evaluated the impact of various doses of γ-irradiation cross-linking on the thermal and mechanical properties of CMC/Ca-montmorillonite (MMT) clay composites for the removal of Cu(II) ions from wastewater [65]. Tensile strength continuously improved with radiation dose up to 10 kGy, beyond which it began to decline. An increase in Ca-MMT clay loading ratio further enhanced tensile strength, whereas elongation at break consistently decreased with increasing clay content and radiation dose. Thermal and structural variations were confirmed through various physicochemical analyses. Heavy metal adsorption studies revealed that the composite irradiated at 10 kGy exhibited a Q_max_ of 54.6 mg/g for Cu(II) ions under optimum conditions (contact time = 300 min, pH = 5). Kinetic modeling indicated that the adsorption data fitted well with the PSO kinetic model, while isotherm analysis revealed that the Cu(II) adsorption mechanism closely followed the Langmuir isotherm model. In addition, El-Arnaouty et al. synthesized CMC-g-methacrylic acid (MAAC)/acrylamide (AAM) hydrogels using γ-irradiation for the removal of pollutants from wastewater [66]. The gel fraction (%) increased with higher AAM concentrations, while the swelling capacity improved with increasing MAAC content in the hydrogel formulation. The diffusion mechanism and swelling kinetics suggested that water diffusion followed a non-Fickian transport mechanism. The findings of the work show that the developed hydrogel had a higher grafting ratio, grafting yield, thermal stability, and swelling behavior than those individually grafted MAAC and AAM onto CMC by this technique. Spectroscopic analysis confirmed the successful grafting of both AAM and MAAC onto CMC. The adsorption capacity of the developed hydrogels was evaluated for dyes, such as methyl green and acid blue, as well as metal ions, including Co(II) and Cu(II).

**Figure 5:**
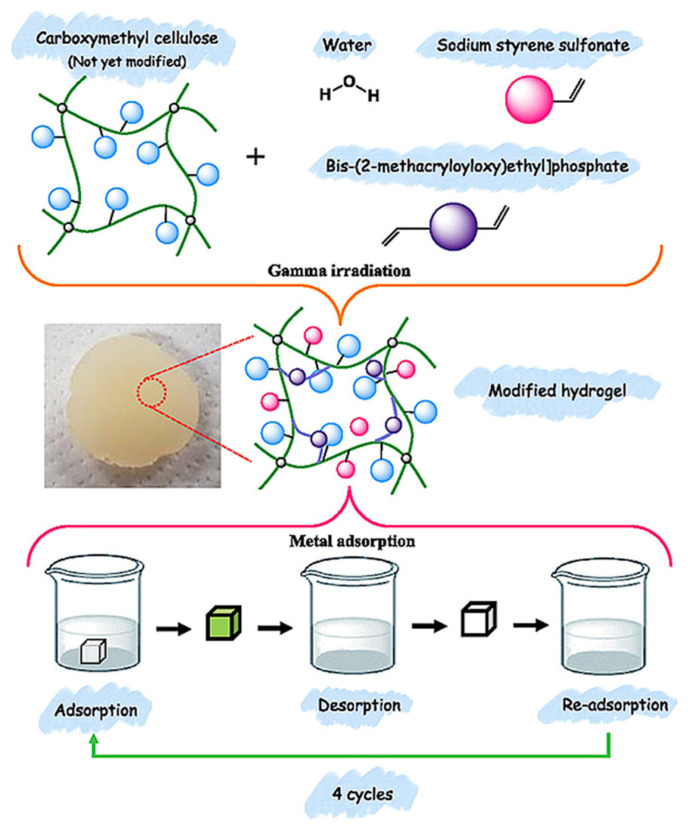
Schematic of irradiation-assisted synthesis and reusability assessment of CMC/SSS/BMEP hydrogels after four cycles. Reproduced from [68] with permission from the Elsevier publishers.

Furthermore, Hong et al. developed radiation-grafted CMC/sodium styrene sulfonate (SSS) hydrogels via γ-irradiation for the removal of metal ions from aqueous solutions [67]. The developed hydrogels have higher mechanical strength with a Poisson’s ratio of 0.45 and compressive modulus of 3.4 (kPa). The presence of phosphonate, sulfonate, hydroxyl, and carboxylate groups was confirmed through spectroscopic analysis. The swelling index increased with higher SSS content, attributed to the presence of hydroxyl and sodium sulfonate groups in the hydrogel structure. Adsorption experiments demonstrated that the hydrogels effectively removed metal ions in the following order: 33.5% of iron (Fe(II)), 35.2% of manganese (Mn(II)), 41.4% of Pb(II), and 68.0% of chromium (Cr(III)). Kinetic studies indicated that the adsorption process was governed by chemisorption, as described by the PSO kinetic model. Furthermore, the monolayer sorption occurred on the surface of the CMC/SSS hydrogel, suggesting the Langmuir isotherm model provides a better fit. The Q_max_ of the hydrogel for Zn(II), Cr(III), and Fe(II) was determined as 30.34, 36.65, and 79.78 μg/g, respectively. Building on their previous work, Hong et al. carried out γ-irradiation induced graft copolymerization of CMC with two active vinyl monomers, bis[2-(methacryloyloxy)ethyl]phosphate (BMEP) and SSS, for the adsorption of heavy metal ions [68]. The swelling capacity and compression moduli of the prepared hydrogels decreased with increasing BMEP concentration. The hydrogels exhibited higher recovery efficiencies for most metal ions, achieving up to 70% recovery for Ni(II). This adsorption performance was attributed to the presence of various active sites—phosphonate, sulfonate, hydroxyl, and carboxylate groups—within the hydrogels. Figure 5 illustrates the schematic of irradiation-assisted synthesis and reusability assessment of CMC/SSS/BMEP hydrogels. After four cycles of adsorption–desorption treatments, the hydrogels maintained a high removal efficiency of approximately 81%, confirming their reusability. These characteristics position the developed hydrogels as promising candidates for sustainable, eco-friendly adsorbents in environmental remediation applications.

Additionally, El-Naggar et al. fabricated sodium alginate (SA) and CMC-based SAB hydrogels using γ-irradiation induced cross-linking for the removal of heavy metal ions from wastewater [69]. The swelling capacity of the hydrogels irradiated at a constant dose of 2.5 kGy increased with rising SA content, whereas the gel fraction (%) decreased with higher SA concentrations. Morphological images showed compatibility between SA and CMC. A similar thermal response was observed in the developed hydrogels with different formulations. Adsorption studies demonstrated that the SA/CMC hydrogels effectively removed heavy metal ions, specifically Fe(III) and Ni(II), from wastewater. These findings suggest that hydrogels hold strong potential for environmental remediation applications.

Furthermore, El-Hag Ali et al. synthesized 2-acrylamido-2-methyl propane sulfonic acid (AMPS) and CMC-based SAB hydrogels via γ-irradiation induced cross-linking and copolymerization for the removal of heavy metals from wastewater [70]. The developed SAB hydrogels showed a tendency to recover metal ions (Co(II), Mn(II), Fe(III), and Cu(II)) from their respective solutions. The results indicated that the binding capability depends on various physicochemical properties, including metal ion concentration, pH, and the internal composition of hydrogels. The binding capacity increased with higher AMPS content, elevated solution pH, and greater metal ion concentrations. Isotherm studies revealed that the adsorption data were best described by the Langmuir isotherm model. Furthermore, the regeneration performance of the AMPS/CMC copolymeric SAB hydrogels remained consistent over five adsorption–desorption cycles, confirming their stability and reusability.

In summary, ionizing radiation-processed cellulose-based hydrogels have been effectively employed for the removal of various heavy metal ions, including as U(VI), F^−^, Pb(II), Zn(II), Co(II), Ag(I), Sr(II), Cu(II), Fe(II), Fe(III), Mn(II), Cr(III), and Ni(II). Findings from multiple studies demonstrate that this technology is suitable for fabricating cellulose-based hydrogels for heavy metal removal.

#### 5.1.2. Removal of Dyes

**Figure 6:**
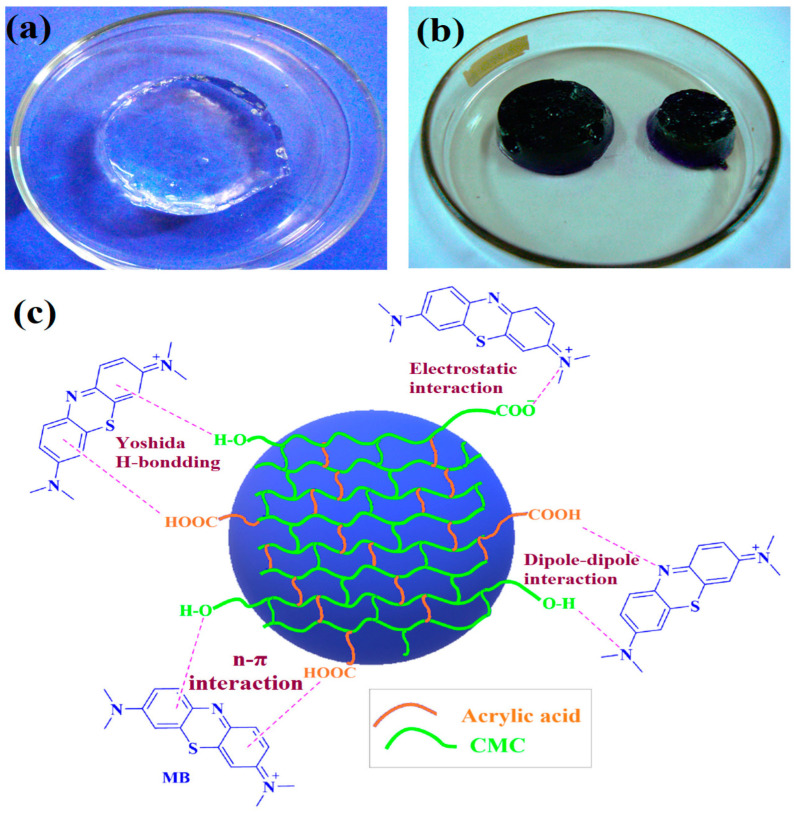
γ-irradiated CMC/PAAC hydrogels (**a**) before (**b**) after dye adsorption (**c**) proposed adsorption mechanism for the MB dye [77].

Dyes are complex compounds that naturally adsorb onto substrate surfaces to impart color, making them valuable for various applications, including pulp, paper, cosmetics, and textiles [71,72]. They are broadly categorized into natural and synthetic types, exhibiting either cationic or anionic properties [73]. Global dye consumption is estimated at 10,000 tons per year and continues to rise due to increasing industrial demand and population growth [74]. However, the discharge of organic dye effluents from textile and other industries poses significant risks to both aquatic ecosystems and human health [75]. In this regard, Elshahawy et al. developed CMC/PAAC hydrogels embedded with zinc oxide (ZnO)/ZnO doped silver (ZnO@Ag) nanoparticles via γ-irradiation for the remediation of lerui acid brilliant blue (LABB) dye [76]. In this work, ZnO and ZnO@Ag nanoparticles were first synthesized and characterized to confirm successful fabrication. Then, impregnated nanoparticles into CMC/PAAC solution, and γ-irradiation exposure assisted in nanocomposites fabrication. The physicochemical characteristics of the prepared hydrogels were studied through various techniques: FTIR, XRD, SEM, TEM, and EDX. FTIR confirmed the structural changes in the CMC. Morphological analysis revealed that the nanoparticles were evenly distributed within the polymeric matrix without aggregation. The swelling characteristics of the CMC/PAAC hydrogels were significantly enhanced by incorporating nanoparticles as reinforcing agents. A maximum decolorization efficiency of 93% was achieved under optimal conditions: pH 4, an initial dye concentration of 50 mg/L, an exposure time of 90 min, and a catalyst dosage of 50 g/L. The nanocomposite hydrogel demonstrated excellent removal of LABB dye from wastewater. However, after five reuse cycles, the decolorization efficiency declined to 60%. These results indicate that nanocomposite hydrogels are both cost-effective and practical for the decolorization of dye-contaminated wastewater. In a related study, Sutradhar et al. synthesized CMC/PAAC hydrogels via γ-irradiation for the removal of methylene blue (MB) dye [77]. The polymeric concentration and radiation doses were optimized. Physicochemical analyses confirmed the successful fabrication of thermally stable hydrogels. The maximum swelling value (18,774.60 g/g) was obtained at a CMC/PAAC composition ratio of 7.5:5. As shown in Figure 6, the hydrogel exhibited a Q_max_ of 681 mg/g for MB at a concentration of 80 mg/L, and a desorption efficiency of 95% was achieved in 2M HCl. Kinetic studies showed a non-uniform physisorption mechanism occurring on a heterogeneous surface, which followed Schott’s PSO kinetic model. The proposed mechanism of MB adsorption within hydrogels is shown in Figure 6. Initially, CMC/PAAC hydrogel swelled considerably and achieved an equilibrium swelling characteristic while retaining its shape. During this, the polymeric network was fully unfolded, and large pathways were created. When the CMC/PAAC adsorbent was placed in an MB solution, the MB molecules diffused into the hydrogel and quickly bonded with hydroxyl and carboxylate groups [78]. The numerous active sites are presented due to the higher swelling of the adsorbent, which leads to the high adsorption capacity [79]. CMC/PAAC adsorbent possesses several electron pairs, while MB is a cationic organic dye with positive charges on its surface, resulting in an electrostatic attraction between the hydrogel and the dye. Moreover, the aromatic rings in the chemical structures of both MB and the adsorbent enhance π-π stacking interactions [80]. These findings suggest that the developed hydrogels are promising candidates for large-scale industrial applications in environmental remediation.

Furthermore, Al-Gorair et al. extracted cellulose nanocrystals (CNC) from pea peels via acid hydrolysis and subsequently fabricated a pectin (PEC)/PAAC/CNC nanocomposite using γ-irradiation for MB dye remediation [81]. Physicochemical analyses confirmed the successful incorporation of CNC into the superabsorbent, resulting in enhanced adsorption and swelling properties. Adsorption kinetic studies indicated that the obtained adsorption data fit well with the Avrami kinetic model, with minimal standard deviation. The nanocomposite hydrogel exhibited a Q_max_ of 576.62 mg/g. The adsorption isotherm of MB by PEC/PAAC/CNC conformed to the Langmuir isotherm model. Thermodynamic studies revealed that the adsorption process was endothermic and spontaneous. Similarly, Hakam et al. synthesized BC/PAAC-based hydrogels by irradiation-grafting AAC onto BC for the removal of MB from aqueous solutions [82]. The morphological images of the developed hydrogels showed a highly porous structure. The equilibrium water content (%) increased with higher AAC concentrations and was sensitive to changes in solution pH. The synthesized hydrogels absorbed MB over time due to the electrostatic interaction between the carboxyl groups of the hydrogels and the imine groups of MB. In another study, Helal et al. synthesized and characterized PVA/CMC/CS-based hydrogels using γ-irradiation for the removal of ionic dyes, specifically direct blue 1 (DB1) and MB, and evaluated their biodegradability [83]. The prepared hydrogels were characterized by determination of swelling (%), gelation (%), SEM, and XRD to ensure successful formation. Adsorption studies revealed that both DB1 and MB conformed to the Langmuir isotherm model. Kinetic analysis indicated that DB1 adsorption followed a PSO kinetic model, whereas MB adsorption adhered to a PFO model. The biodegradation experiment demonstrated that the pure hydrogels degraded more rapidly in soil than those that had adsorbed DB1 and MB. Regarding soil moisture retention, the hydrogels synthesized at 20 kGy exhibited the highest water-holding capacity in sandy soil (SS), while those prepared at 2.5 kGy showed the lowest performance.

In addition, Liu et al. developed a smart hydrogel based on N-isopropyl acrylamide (NIPAAM)/high-substituted hydroxypropyl cellulose (HPC)/graphite carbon nitride (g-C_3_N_4_) using EB pre-radiation polymerization and cross-linking methods, imparting both photocatalytic and thermoresponsive properties [84]. The uniform dispersion of g-C_3_N_4_ nanosheets within the hydrogel matrix enhanced its photocatalytic performance under visible light. When applied for the adsorption–photocatalytic removal of rhodamine B dye in an aqueous medium under visible light, the hydrogel achieved a removal efficiency of 71.4%, which increased with the mass ratio of g-C_3_N_4_. The hydrogel also exhibited a thermal shrinkage ratio of 90.6% after 5 min at 60 °C and demonstrated a self-recovering, recycling-free function suitable for use in portable photocatalytic reaction devices. These highly photocatalytic properties and thermally driven recycling-free characteristics of the hydrogel-based photocatalyst highlight its potential as a promising multifunctional material for wastewater treatment applications.

Similarly, Gad et al. fabricated titanium dioxide (TiO_2_)/CMC-based composite hydrogels using γ-irradiation for the removal of basic violet 7 dye [17]. The tensile strength of the hydrogels increased with the addition of TiO_2_ and higher irradiation doses, attributed to improved compatibility between the CMC matrix and the TiO_2_ filler. In contrast, elongation at break decreased with increasing TiO_2_ content and irradiation dose. The γ-irradiation cross-linked hydrogels also demonstrated enhanced thermal stability. Under optimal conditions, the hydrogels exhibited a Q_max_ of 123.6 mg/g for violet 7 dye. The adsorption kinetics closely followed the PSO kinetic model, while isotherm studies indicated that the dye adsorption conformed to the Langmuir model, suggesting chemical monolayer adsorption. These findings highlight the potential of TiO_2_/CMC composite hydrogels for large-scale industrial dye removal applications. Furthermore, Taleb et al. synthesized CMC/poly(vinyl alcohol) (PVA)-based copolymeric hydrogels using EB-irradiation for the removal of dyes [85]. Thermodynamic parameters indicated that the adsorption process was exothermic, primarily driven by electrostatic interactions. The adsorption performance improved with increasing CMC content and followed the order: direct pink 3B > acid green B > ismative violet 2R. However, adsorption capacity decreased with rising pH levels. Isotherm studies revealed that the adsorption data followed the Freundlich model. These results indicate that irradiation cross-linked CMC/PVA copolymeric hydrogels hold significant potential for dye removal applications.

In summary, ionizing radiation-processed cellulose-based hydrogels have been effectively employed to remediate various dyes, including LABB, MB, rhodamine B, basic violet 7 dye, direct pink 3B, acid green B, and ismative violet 2R. Findings from multiple studies highlight the potential of this technology for fabricating cellulose-based hydrogels for dye removal applications.

#### 5.1.3. Removal of Organic Contaminants

Organochlorines, polyphenols, and aromatic compounds are considered among the most hazardous pollutants to the ecosystem. These contaminants originate from various sources, including the leaching and chemical industries, runoff from forest and agricultural lands (through intensive herbicide and pesticide use), industrial effluents, and discharge from aerial applications [86,87]. Their remediation can be achieved through various processes, including sorption, catalysis, and filtration. In hydrogel-based systems, pollutant adsorption occurs through attachment to available functional groups [88]. Cellulose-based hydrogels have gained attention due to their low cost and minimal environmental impact [89]. In this regard, Abdel Ghaffar et al. developed CMC-based hydrogels using γ-irradiation for the removal of organic contaminants such as 2,4-dichlorophenoxyacetic acid (2,4-D) and 4-chlorophenol [90]. Hydrogel derivatives were synthesized by grafting MAAC individually onto CMC to produce P(CMC/MAAC), and by co-grafting MAAC with AAM to form P(CMC/MAAC/AAM). The physicochemical properties of the prepared hydrogels were analyzed using several techniques. The thermal analysis revealed that the binary system has higher thermal stability as compared to blank hydrogels. FTIR analysis confirmed the grafting of both monomers onto CMC. XRD showed higher intensity of diffraction peaks, which might be due to enhanced ordering of the chains owing to cross-linking induced by γ-irradiation and H-bonding interaction between functional groups of CMC and monomers, leading to higher crystallinity of the binary system. Also, it exhibited higher swelling capacity and gel fraction (%) than the singly grafted hydrogel. However, the adsorption capacity of the singly grafted hydrogel for 2,4-D and 4-chlorophenol exceeded that of the binary system. Both hydrogels demonstrated greater adsorption efficiency for 2,4-D compared to 4-chlorophenol. In a related study, Abdel Ghaffar et al. grafted MAAC and AAM individually onto CMC via γ-irradiation to develop P(CMC/MAAC) and P(CMC/AAM) hydrogels for the removal of hazardous water pollutants [91]. In both cases, grafting ratio and grafting yield increased with rising monomer concentration, with P(CMC/AAM) showing higher values. FTIR analysis confirmed the grafting of both monomers onto CMC. The thermal analysis showed that the prepared hydrogels had high thermal stability. The SEM micrographs revealed that CMC performed as a backbone in the P(CMC/MAAC) hydrogel to provide strength, whereas MAAC contributed to enhancing pore size. The swelling capacity of P(CMC/MAAC) increased with higher MAAC content, while that of P(CMC/AAM) decreased with increasing AAM content due to greater cross-linking density. The diffusion mechanism and swelling kinetics indicated that water diffusion followed a non-Fickian transport mechanism. The adsorption capacities of the synthesized hydrogels were evaluated for a range of pollutants, including organic contaminants (e.g., 2,4-D and 4-chlorophenol), dyes (e.g., methyl green and acid blue), and heavy metals (Co(II) and Cu(II)). Based on these findings, the study demonstrates the potential of these hydrogels as a viable strategy for the removal of diverse pollutants from wastewater. In a separate research, Kimura et al. dissolved high concentrations of cellulose in room-temperature ionic liquids (RTILs), specifically N,N-diethyl-N-methyl-N-(2-methoxyethyl)ammonium (DEMA)-formate and 1-ethyl-3-methylimidazolium (EMI)-acetate with an 18 wt% water content, followed by γ-irradiation to produce cellulose hydrogels [92]. Dynamic viscoelasticity studies revealed that after irradiation, the storage modulus curve of the 20 wt% cellulose in EMI-acetate was almost constant, while that of the loss modulus increased with frequency. This confirms that the after-irradiation sample transforms into gel at 353 K. GPC analysis showed shifting of retention times with the increase in irradiation dose. At an irradiation dose of 10 kGy, gel fractions of 19% and 13% were achieved for DEMA-formate and EMI-acetate, respectively. The gel fractions (%) were influenced by several factors, including water content, initial cellulose concentration, and irradiation temperature. The obtained hydrogel readily absorbed water, ethanol, methanol, N,N-dimethylacetamide, dichloromethane, and RTILs.

In summary, ionizing radiation-processed cellulose-based hydrogels have been employed to remediate organic contaminants, including 2,4-D, 4-chlorophenol, ethanol, methanol, N,N-dimethylacetamide, dichloromethane, and RTILs. However, the current literature on this topic is limited; therefore, further research is needed to fully assess the potential of this technology for fabricating cellulose-based hydrogels for organic contaminant remediation.

**Table 3:** Ionizing radiation processed cellulose-based hydrogels for environmental remediation.

Adsorbent Constituents	Fabrication Technology (Ionizing Radiation)	Potential for Adsorbate Removal, Adsorption Kinetics, Isotherms, and Regeneration Efficiency	Applications	References
Cellulose/Urea/NaOH/AAM	γ (dose rate: 5 kGy/h)	449.5 mg/g of U(VI), desorption rate 87.1%, PSO kinetic model, Langmuir model	Potential for wastewater treatment	[59]
Zr(IV) immobilized CMC/MW-CCNTs	EB absorbed dose: 5–30 kGy, (dose rate: 5 kGy/pass)	36.657 mg/g of F^−^, PSO kinetic model, Freundlich and Langmuir models	Selectivity for F^−^ removal from the hydrometallurgical system and water body	[60]
**Removal of heavy metals**				
HEC/AAC	γ absorbed dose: 10, 20, 30, 40, and 50 kGy, (dose rate: 0.7 kGy/h)	26.65 mg/g of Pb(II), PSO kinetic model, Freundlich isotherm	Pb(II) removal	[62]
CYANEX 471X/HEC/AAC	γ absorbed doses: 10, 20, 30, 40, and 50 kGy, (dose rate: 0.9 kGy/h)	12 mg/g of Ag(I), maximum desorption 70%, non-Fickian diffusion, Five cycles	Ag(I) ions capture from acidic nitrate medium	[63]
CMC/Ca-MMT clay	γ absorbed doses: 5, 10, and 15 kGy, (dose rate: 0.33 Gy/s)	54.6 mg/g of Cu(II), PSO kinetic model, Langmuir isotherm model	Removal of Cu(II) ions from wastewater	[65]
Cellulose/MAAC/AAM	γ absorbed dose: 20 kGy, (dose rate: 0.74 Gy/s)	Metal ions (Cu(II) and Co(II)), Dyes (acid blue dye and methyl green), Non-Fickian transport mechanism	Removal of pollutants from wastewater	[66]
CMC/SSS/BMEP	γ absorbed doses: 10–100 kGy	70% for Ni(II), adsorption-desorption performance (~81%), Four cycles	Heavy metal ions adsorption	[68]
CMC/SSS	γ absorbed doses: 20 to 100 kGy	79.78 μg/g of Fe(II), 3.60 µg/g of Pb(II), and 36.65 µg/g of Cr(III), PSO kinetic model, Langmuir isotherm model	Removal of metal ions from aqueous solutions	[67]
CMC/SA	γ absorbed dose: 20 kGy, (dose rate: 6.92 kGy/h)	Heavy metals (Ni(II) and Fe(III))	Heavy metal ions from wastewater	[69]
CMC/AMPS	γ absorbed dose: 10–20 kGy, (dose rate: 10.28 kGy/h)	Heavy metals (Co(II), Mn(II), Fe(III), and Cu(II)), Langmuir isotherm model, Five cycles	Removal of heavy metals from model wastewater	[70]
CMC/HPMCP/CM-CS	γ absorbed dose: 40 and 80 kGy (dose rate: 20 Gy/min)	Sr(II) removal: 83.3 mg/g for HPMCP, 99.0 mg/g for CMCS, and 108.7 mg/g for CMC	Adsorption and desorption of Sr(II) ions	[64]
**Removal of dyes**				
CMC/AAC/ZnO/ZnO@Ag	γ absorbed doses: 10–30 kGy, (dose rate: 0.614 kGy/h)	Decolorization efficiency 93% of LABB dye, 60% performance after five cycles	Efficient photocatalytic remediation	[76]
NIPAAM/Substituted HPC/g-C_3_N_4_	EB absorbed dose: 25 kGy, (dose rate: 5 kGy/pass)	Adsorption–photocatalytic removal of rhodamine B 71.4%	Promising new material with extensive applications in wastewater treatment	[84]
CMC/AAC	γ absorbed doses: 1–15 kGy, (dose rate: 3 kGy/h)	681 mg/g of MB, Desorption efficiency 95%, Schott’s PSO model	Dye removal	[77]
CNC/PEC/AAC	γ absorbed dose: 20 kGy	576.62 mg/g of MB, Avrami kinetic model, Langmuir isotherm model	Remediation of basic dyes	[81]
CMC/TiO_2_	γ absorbed doses: 5, 10, and 15 kGy, (dose rate: 0.33 Gy/s)	123.6 mg/g of violet 7 dye, PSO kinetic model, Langmuir model	Removal of basic dye from wastewater	[17]
BC/AAC	γ absorbed dose: 30 kGy	MB	Removal of MB dye in aqueous solution	[82]
PVA/CMC	EB absorbed doses: 5–20 kGy	Dye removal order: Direct pink 3B > acid green B > ismative violet 2R, Freundlich model	Removal of dyes	[85]
**Removal of organic contaminants**				
MAAC/CMC	γ absorbed doses: 20 kGy, (dose rate: 0.74 Gy/s)	4-chlorophenol and 2,4-D	Removal of organic contaminants	[90]
CMC/AAM/MAAC	γ absorbed dose: 20 kGy, (dose rate: 0.74 Gy/s)	Organic contaminants (2,4-D and 4-chlorophenol), dyes (methyl green and acid blue dye), and heavy metals (Co(II) and Cu(II)), Non-Fickian transport mechanism	Removal of hazardous water pollutants	[91]

## 6. Agricultural Applications

In agriculture, cellulose hydrogels are used for soil conditioning and controlled release of agrochemicals. A comprehensive summary of the agricultural applications of ionizing radiation-processed cellulose-based hydrogels is presented in Table 4.

El-diehy et al. developed poly(acrylamide) (PAAM)/CMC-based SAB hydrogels using γ-irradiation to improve the drought tolerance of *Beta vulgaris* [93]. The hydrogels exhibited a maximum swelling capacity of approximately 500 g/g, which was influenced by both the AAM content and radiation dose. Morphological analysis revealed a porous structure that facilitated water absorption. The irradiation-modified hydrogels demonstrated superior water retention, retaining about 75% of absorbed water after 16 days, compared to a 50% loss within 5 days in the unmodified samples. Under severe drought conditions, the application of the developed hydrogels increased root length by 32%, shoot length by 18%, shoot dry weight by 15%, and shoot fresh weight by 15%. Consequently, protein content increased by 19%, yield by 22%, and carbohydrate levels by 13%. A reduction in oxidative damage was observed, as indicated by increased leaf chlorophyll content and decreased activity of stress-related enzymes. Overall, the developed eco-friendly PAAM/CMC hydrogels demonstrate significant potential for addressing agricultural challenges associated with water scarcity.

**Figure 7:**
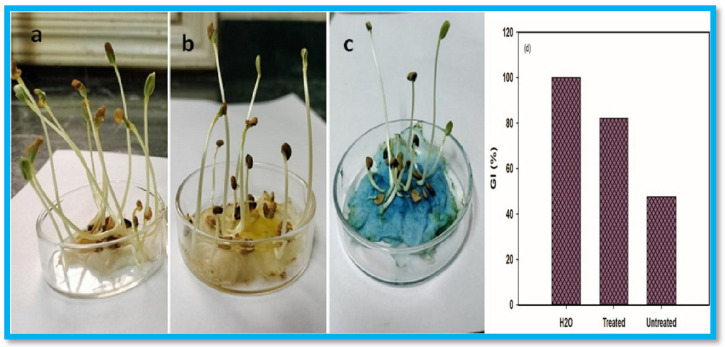
Illustration of the growth of fenugreek seeds over the week using mediums (**a**) deionized water, (**b**) treated MB dye, and (**c**) untreated MB dye; (**d**) GI(%). Reproduced from [95] with permission from Elsevier publishers.

Similarly, Zhao et al. developed polyampholytic (CMC/CS) hydrogels using EB irradiation at room temperature [94]. The incorporation of CS improved both the mechanical strength and gel fraction (%) of the hydrogel blends compared to pure cross-linked CMC. These polyampholytic (CMC/CS) hydrogels exhibited pH sensitivity and high water absorption capacity. Moreover, due to the biodegradable nature of CS, the hydrogels were susceptible to enzymatic biodegradation. Consequently, the CMC/CS hydrogels synthesized via EB-irradiation present promising biodegradable materials with potential applications in various fields. Similarly, Mohamed et al. fabricated HEC/PVA/cuprous oxide (Cu_2_O)-rGO/bismuth vanadate (BiVO_4_)-based nanocomposites using EB irradiation for the visible-light-driven decolorization of MB dye [95]. The highest gelation degree was achieved by optimizing the fabrication process. The incorporation of rGO enhanced the MB decolorization efficiency by 20% compared to nanocomposites without rGO. The presence of BiVO_4_ yielded 90% decolorization efficiency at pH 11 after 150 min using a (10 ppm) MB solution. The nanocomposite maintained 71% efficiency after five reuse cycles. The treated dye solution exhibited a germination index (GI) of 82% on Fenugreek seeds, as shown in Figure 7, indicating its suitability for irrigating gardens and playgrounds.

**Figure 8:**
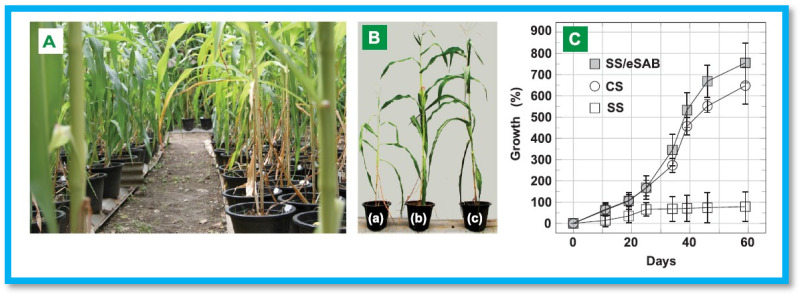
(**A**) Images of baby corn grown in various soils, (**B**) Efficiency in (**a**) SS (**b**) SS/SAB (**c**) cultivated soil at 60 days, (**C**) Graph of growth (%) vs number of days. Reproduced from [96] with permission from Elsevier publishers.

Furthermore, Piroonpan et al. developed PAA/sugarcane bagasse cellulose (CSB)-based interpenetrating polymer networks using EB-irradiation induced simultaneous cross-linking and free radical graft copolymerization for SS amendment [96]. The CSB/PAA hydrogels were prepared in two different systems: water and aqueous sodium hydroxide (NaOH)/urea co-solvent. The latter system, designated as SAB, exhibited superior swelling behavior, reaching a maximum capacity of approximately 400 g/g. For pot experiments, baby corn was used as a model plant to assess the performance of SAB for SS amendment. The results revealed enhanced water retention and reduced water flow rate in SS amended with SAB. Moreover, the SAB-treated soil improved baby corn growth and overall product quality, as illustrated in Figure 8. These findings indicate that SAB has potential for altering SS and improving cultivation in drought-prone areas.

Similarly, in a study involving MMT clay, Salmawi et al. synthesized CMC/AAC/MMT clay-based SAB hydrogels using γ-irradiation [97]. Physicochemical analyses were conducted to assess several properties of the developed hydrogels. The hydrogels exhibited a higher swelling index in distilled water compared to salt solutions, and greater swelling was observed in the basic medium than in the acidic medium. MMT clay content had a reciprocal impact on the water retention properties of the hydrogels. These findings suggest that the synthesized hydrogels can be used for water management in horticulture and agriculture, particularly in drought-prone and sandy areas.

### Release of Agrochemicals

**Figure 9:**
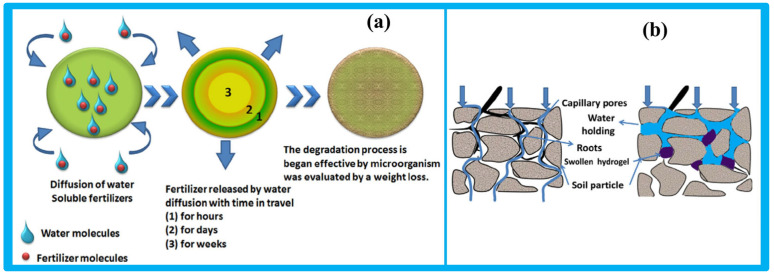
(**a**) The depiction of the release of fertilizer from the hydrogel, (**b**) the influence of the hydrogel on the soil surface for the growth of plants. Reproduced from [98] with permission from Elsevier publishers.

The underlying mechanism of hydrogel function in agriculture is illustrated in Figure 9. Following rainfall or irrigation, water infiltrates the soil and drains downward due to gravity. Although such soils may have good aeration, they often lack adequate water retention. Cellulose-based hydrogels address this limitation by acting as soil conditioners, retaining moisture, and creating favorable conditions for plant growth [98].

In this regard, Elbarbary et al. prepared CMC/poly(vinylpyrrolidone) (PVP)-based SAB hydrogels using γ-irradiation for controlled release of fertilizers [99]. The hydrogels exhibited improved water retention and swelling capacity with increasing CMC content. The swelling properties of the hydrogels were attained within just 20 min. Monopotassium phosphate (MPK), urea, and nitrogen–phosphate–potassium (NPK) fertilizers were incorporated into the hydrogel to supply essential nutrients, including potassium, nitrogen, and phosphorus. Water retention (%) ranged from 28% to 36% during the first 72 h, followed by a slower release phase lasting up to 9 days. The hydrogels demonstrated lower swelling in fertilizer solutions compared to distilled water. The slow release of fertilizers was primarily governed by the adsorption–desorption mechanism of the hydrogels. Among the incorporated fertilizers, urea was released approximately 10 times faster than phosphate-based compounds. To assess their applicability in agricultural fields, the impact of CMC/PVP hydrogels on seedling growth was evaluated using *Zea mays* seeds. The soil mixed with fertilizer-loaded CMC/PVP hydrogels showed a higher growth rate compared to the untreated soil. The enhanced swelling capacity, slow fertilizer release, and gradual water retention properties of the developed hydrogels highlight their potential as effective soil conditioners and controlled-release systems for agricultural applications.

Furthermore, Maziad et al. developed CMC/PAAM and CMC/PAAM/silica (Si)-based SAB hydrogels using γ-irradiation for the controlled release of some agrochemicals [6]. Among the synthesized hydrogels, the CMC/PAM/Si composite exhibited the maximum swelling ratio of 12,000% at pH 12 with 5% Si content. Potassium nitrate (KNO_3_) was loaded into the hydrogels to evaluate their release behavior. The results indicated that KNO_3_ release was closely associated with the swelling properties of the hydrogels, a key attribute influencing their release kinetics. The KNO_3_ release was reliant on the constituent ratios, network chain density, matrix swelling, and pore size. Cumulative release studies showed that KNO_3_ release increased with higher loading percentages. Kinetic analysis revealed a non-Fickian diffusion mechanism, which shifted to a Fickian mechanism upon Si incorporation. These findings suggest that the developed hydrogels are promising candidates for agricultural applications, particularly in regulating agrochemical release to support plant growth. Similarly, Ghobashy et al. prepared AAM/HEC-based ultra-absorbent hydrogel (UAH) using γ-irradiation for use as a soil conditioner [100]. The study addresses the potential detrimental effects of climate variability on water reservoirs, particularly water for agricultural purposes. The developed PAAM/HEC hydrogel exhibited a maximum swelling capacity of 23.4 g/g. Alkaline hydrolysis significantly enhanced water absorbency, with potassium hydroxide treatment achieving the highest capacity (1220 g/g), followed by NaOH (622 g/g) and lithium hydroxide (540 g/g). In-vitro release analysis showed controlled and slow release of fertilizers from UAH, with full release of the urea occurring over 22 days. The UAH retained water for up to 28 days and effectively supported the growth of *Zea mays* L. at various drought-induced stress levels of 0%, 25%, 50%, and 100%, increasing shoot length by 16%, 19%, 24%, and 20%, respectively. Moreover, UAH enhanced the carotenoid and chlorophyll (a, b, and a + b) contents of the maize plant leaves. These findings suggest that the developed hydrogels are promising candidates for optimizing water and fertilizer management in maize cultivation under drought conditions.

In another study, Raafat et al. developed CMC/PVP-based SAB hydrogels using γ-irradiation for agricultural applications [101]. The effects of polymer composition and radiation dose were investigated using several techniques. Urea as a fertilizer model was loaded onto the CMC/PVP SAB hydrogel to deliver nitrogen nutrients. The developed hydrogels exhibited favorable swelling behavior, which was influenced by both the absorbed radiation dose and polymer composition. Additionally, the swelling response was sensitive to cationic and ionic strength. The good water retention capacity and slow urea release make these hydrogels suitable for agricultural applications. Similarly, El-Naggar et al. developed CMC-based films incorporated with fertilizers and metal salts using the solution casting method, followed by exposure to γ-irradiation, and characterized specific properties [102]. The highest gel fraction (%) was achieved at an absorbed dose of 10 kGy. Physicochemical analyses confirmed the successful incorporation of additives and enhanced mechanical properties. The complex formation was attributed to the coordination between the carboxylate group of CMC and the metal cation. In another study, Wach et al. synthesized dual stimuli (temperature and pH)-responsive HPC/CMC hydrogels via γ-irradiation, with and without a cross-linking agent (poly(ethylene glycol) diacrylate (PEGDA)), for potential applications such as nutrient delivery depots [103]. The swelling behavior of the hydrogels was influenced by both stimuli: the pH, owing to the anionic functional groups of CMC, and temperature, attributed to the amphiphilic character of HPC. Hydrogels with equal concentrations of HPC and CMC exhibited the highest swelling capacity. At pH 2, the hydrogels exhibited a 95% reduction in swelling, indicating strong pH responsiveness. Similarly, a 70% decrease in swelling occurred when the temperature rose to 55 °C, confirming thermal sensitivity. These findings suggest that the developed hydrogels are beneficial in featured applications such as water reservoirs for biodegradable microelements and nutrient delivery depots.

**Table 4:** Ionizing radiation processed cellulose-based hydrogels for agricultural applications.

Superabsorbent Constituents	Fabrication Technology (Ionizing Radiation)	Applications	References
CMC/AAM	γ absorbed doses: 10, 20, 30, 40, and 50 kGy, (dose rate: 0.68 kGy/h)	For enhancing *Beta vulgaris* under drought stress	[93]
PVA/CMC/CS	γ absorbed doses: 2.5, 5, 10, and 20 kGy, (dose rate: 2.5 kGy/h)	Water-holding capacity and water-retention of sandy soils	[83]
HEC/PVA/Cu_2_O-rGO/BiVO_4_	EB absorbed doses: 25, 35, and 45 kGy	Suitability for the irrigation of gardens and playgrounds	[95]
CSB/PAAC	EB absorbed doses: 5–100 kGy	SS amendment	[96]
HEC/AAM	γ absorbed doses: 10, 20, 30, 40, and 50 kGy, (dose rate: 0.83 kGy/h)	Maize planting in drought conditions	[100]
CMC/AAC/Ca-MMT clay	γ absorbed doses: 2.5,5, 7.5, and 10 kGy	Potential as water-managing material for agriculture and horticulture in desert and drought-prone areas	[97]
CMC/PVP	γ absorbed doses: 20, 25, and 30 kGy	Agriculture applications	[101]
CMC/PVP	γ absorbed doses: 5, 10, 20, and 30 kGy, (dose rate: 2.05 kGy/h)	Controlled release fertilizers	[99]
CMC/PAAM/Si	γ	Controlled release of some agrochemicals	[6]
HPC/CMC/PEGDA	EB absorbed doses: 5–100 kGy; (dose rate: 5 kGy/min ± 10%)	Water reservoirs for biodegradable microelements and nutrient delivery depots	[103]

## 7. Conclusions, Limitations, and Future Perspectives

This review highlights the potential of ionizing radiation-processed cellulose-based SAB hydrogels for both environmental and agricultural applications. In environmental contexts, these cellulose-based hydrogels exhibit pollutant adsorption capabilities and regenerative properties. However, current studies predominantly focus on adsorption performance across multiple reuse cycles, often neglecting the impact of regeneration on the mechanical strength and morphology of the adsorbents—which are key factors in evaluating their long-term reusability. Additionally, most research has conducted adsorption experiments using only one or two types of heavy metals/dyes/organic contaminants, thus limiting the ability to assess the adsorbent’s selectivity. Since contaminated water typically contains complex compounds, which are a combination of several pollutants, evaluating the efficacy of cellulose-based hydrogels under multi-contaminant conditions is essential. Expanding the application of these materials to target a broader spectrum of contaminants could enhance their practical relevance in environmental remediation.

In agricultural applications, cellulose hydrogels primarily serve as soil conditioning agents and carriers for the controlled release of agrochemicals to enhance crop productivity. Cross-linking cellulose via ionizing radiation produces hydrogels with a three-dimensional network structure capable of absorbing and retaining large volumes of water. This increased water availability, coupled with reduced evaporation losses, significantly improves soil water retention. However, the water-holding capacity of these hydrogels is highly sensitive to pH variations, and the incorporation of inorganic fertilizers substantially diminishes their water absorption efficiency. Recently, enhancing the biodegradability, mechanical strength, and biocompatibility of hydrogel systems has become a priority in agricultural applications. Despite certain limitations, including insufficient tensile strength, limited load-bearing capacity, suboptimal fertilizer loading/release profiles, and commercialization challenges, focused and targeted research remains essential. Future studies should aim to develop cost-effective, biodegradable hydrogels with improved hydrophobicity, optimized fertilizer loading/release characteristics, and soil-degradable coatings to address these challenges and support the large-scale adoption of hydrogel technologies in agriculture. Furthermore, comprehensive field testing under diverse environmental conditions is crucial to validate laboratory findings and ensure practical applicability.

Cellulose-based hydrogels fabricated through ionizing radiation hold great potential across various applications. This technology needs a huge investment, which is the reason for not full utilization. The main expenses involved in ionizing radiation processing are the acquisition of a γ-irradiator or an EB accelerator and the maintenance of the irradiation facility. Besides the initial investment, additional costs include process monitoring, auxiliary equipment, material handling systems, control systems, acquiring appropriate radiation shielding, and building construction. The authorization to meet the regulatory requirements governing nuclear-related activities is also essential. Continued research and development are essential to fully realize their capabilities and address existing challenges. Ongoing efforts aim to enhance their functionality and broaden their applications. The use of ionizing radiation in hydrogel fabrication has several advantages, including reduced use of toxic chemicals and precise control over the properties of the hydrogels. However, key challenges remain, such as the high cost of radiation equipment and the need for specialized facilities. Future studies should focus on optimizing radiation doses and exploring novel applications in emerging fields.

This review aims to assist both academic researchers and industry experts in designing and developing cellulose-based hydrogels, which have gained significant relevance in environmental remediation and agricultural applications.

## Figures and Tables

**Table 1:** Comparison of characteristics for cellulose/BC/CMC.

Characteristics	Cellulose	BC	CMC
Sources	Cell wall of oomycetes, algae, and plants	*Gluconacetobacter*, *Genera Agrobacterium*, and *Sarcina*	CMC is a derivative of cellulose; however, the synthesis of CMC has been reported from wood residue, paper sludge, mixed office waste, textile waste, and terry towel waste
Synthesis route	−	−	Sodium hydroxides and sodium monochloroacetic acid
Solubility in water	Soluble	Insoluble	Insoluble
Availability	Abundance	Less abundant	Abundance
Mechanical properties	Moderate strength	Superior mechanical strength	Moderate strength
Toxicity	Non-toxic	Non-toxic	Non-toxic
Applications	Biomedical, textiles, electronics, and industrial	Medicines, food, industrial and commercial products	Absorbent, hydrogel, targeted delivery

**Table 2:** Comparison between γ and EB irradiation.

Parameter	γ-Irradiation	EB
Origin	Radioactive source (Co-60)	Beams of electrons (generator/accelerator)
Processing time	Slow (hours)	Fast (seconds to minutes)
Dose delivery	Slow	Quick
Penetration depth	Deeper (excellent penetration)	Less deep (typically <5 cm; depends on energy and density)
Dose rate	Low	High
Direction	Non-directional (omnidirectional)	More directional (controllable)
Speed of irradiation	Slow pace	High speed
Capital investment	High (facility + source management)	High (accelerator + shielding)
Materials Preference	Bulky and high-density materials	Low-to medium-density, thin products
Environmental impact	Radioactive source management is required	No radioactive waste, only electricity is required
Dose uniformity	Very good (especially for complex shapes)	Good (best for simple or thin products)
Safety	Strict due to radioactive material, challenging waste disposal	Easier operation, no radioactive source, simpler regulation
Flexibility	Continuous emission, cannot be turned off instantly	Can be turned on/off instantly, adjustable dose

## Data Availability

No new data were created or analyzed in this study.
